# Novel long noncoding RNA LINC02820 augments TNF signaling pathway to remodel cytoskeleton and potentiate metastasis in esophageal squamous cell carcinoma

**DOI:** 10.1038/s41417-022-00554-2

**Published:** 2022-11-10

**Authors:** Jing Wang, Tie-Jun Huang, Yan Mei, Fei-Fei Luo, De-Huan Xie, Li-Xia Peng, Bao-Qi Liu, Mei-Ling Fan, Jiang-Bo Zhang, Shu-Tao Zheng, Chao-Nan Qian, Bi-Jun Huang

**Affiliations:** 1 Department of Experimental Research, State Key Laboratory of Oncology in South China, Collaborative Innovation Center for Cancer Medicine, Sun Yat-Sen University Cancer Center, Guangzhou, People’s Republic of China; 2 Department of Nuclear Medicine, The Second People’s Hospital of Shenzhen, Shenzhen, People’s Republic of China; 3 Department of Nasopharyngeal Carcinoma, State Key Laboratory of Oncology in South China, Collaborative Innovation Center for Cancer Medicine, Sun Yat-sen University Cancer Center, Guangzhou, People’s Republic of China; 4 Clinical Medical Research Institute, The First Affiliated Hospital of Xinjiang Medical University, State Key Laboratory of Pathogenesis, Prevention and Treatment of High Incidence Diseases in Central Asian, Guangzhou, People’s Republic of China; 5 Guangzhou Concord Cancer Center, Guangzhou, People’s Republic of China

**Keywords:** Cancer, Metastasis

## Abstract

Esophageal squamous cell carcinoma (ESCC) is one of the most common malignant tumors in China. However, there are no targets to treat ESCC because the molecular mechanism behind the cancer is still unclear. Here, we found a novel long noncoding RNA LINC02820 was upregulated in ESCC and associated with the ESCC clinicopathological stage. Through a series of functional experiments, we observed that LINC02820 only promoted the migration and invasion capabilities of ESCC cell lines. Mechanically, we found that LINC02820 may affect the cytoskeletal remodeling, interact with splice factor 3B subunit 3 (SF3B3), and cooperate with TNFα to amplify the NF-κB signaling pathway, which can lead to ESCC metastasis. Overall, our findings revealed that LINC02820 is a potential biomarker and therapeutic target for the diagnosis and treatment of ESCC.

## Introduction

Esophageal cancer (ESCA), which includes esophageal squamous cell carcinoma (ESCC) and esophageal adenocarcinoma (EA), is one of the most common malignancies in the world [1–4]. In China, almost 90% of ESCA patients are diagnosed with ESCC [5]. ESCC develops in the esophageal epithelial mucosa, with smoking and alcohol consumption being significant risk factors [6, 7]. In recent years, ESCC patient prognosis is still poor even though a multidisciplinary approach is used for esophageal cancer treatment [8]. Ultimately, the research on the molecular mechanisms of ESCC is still not clear.

Long noncoding RNAs (lncRNAs) are transcripts longer than 200 nucleotides [9]. Many studies have found that abnormal expression of lncRNAs played an important role in tumorigenesis and tumor metastasis [10]. It can function at the transcriptional level, post-transcriptional level, and epigenetic level [11]. For example, in ESCC, lncRNA MNX1-AS1 can affect proliferation and metastasis [12], while lncRNA FAM83H-AS1 is associated with differentiation and lymph node metastasis [13]. And lncRNA CCAT1 may be a potential therapeutic target for ESCC [14]. Although researchers have found that numerous lncRNAs are abnormally regulated in ESCC, these targets are not used in clinics. In our study, we aim to find more molecular targets for ESCC. Therefore, three paired ESCC tissues and adjacent normal tissue were collected to identify differentially expressed lncRNAs by transcriptome sequencing. Subsequently, we found a novel lncRNA in ESCC, named LINC02820 in the database, and the other names and sequences were shown in Supplementary Table 1. And as far as our knowledge, our study is the first to evaluate the role of LINC02820 in ESCC.

The classical NF-κB signaling pathway plays an important role in malignant tumor progression [15, 16]. The activation signal activates the IKKα/β/γ complex and further phosphorylates the Ser32/36 residues of the IκBα subunit, and then the p50/p65 is released and p65 rapidly underwent nuclear function [17–20].

In the present study, we did a series of functional experiments and found that LINC02820 accelerates the ability of ESCC cell lines to metastasize in vivo and in vitro. Then, combined with the KEGG analysis, we proved that LINC02820 can cooperate with TNFα to amplify the NF-κB signaling pathway. Finally, by the RNA pull-down assays, we found that LINC02820 may interact with splice factor 3B subunit 3 (SF3B3) to function. Overall, our findings revealed that LINC02820 is a potential biomarker and therapeutic target for the diagnosis and treatment of ESCC.

## Materials and methods

### Clinical samples

The First Affiliated Hospital of Sun Yat-Sen University (FAHSYSU) and the Sun Yat-Sen University Cancer Center (SYSUCC) supplied 86 ESCC tumor tissues and adjacent normal tissues (Supplementary Table 2), and 42 of them had prognostic follow-up information (Supplementary Table 2). RNA was extracted from samples and frozen at −80 °C. All human tissue samples were supported by the Medical Ethical Committee of the FAHSYSU and SYSUCC. Informed consent was obtained for all patients.

### Cell culture

The immortalized normal esophageal epithelial cell line (NE1) and 9 human ESCC cell lines KYSE30/K30, KYSE140/K140, KYSE150/K150, KYSE180/K180, KYSE410/K410, KYSE510/K510, KYSE520/K520, EC18, and EC109 (Supplementary Table 3) were kindly gifted by professor Guan (Department of Clinical Oncology, the University of Hong Kong). All cells were cultured in DMEM (Gibco, USA) supplemented with 10% fetal bovine serum (Gibco, USA) and add with a 1% mixture of penicillin and streptomycin. All cells were cultured at 37 °C with 5% CO_2_.

### Isolation of nuclear, cytoplasmic, total RNA, and real-time PCR (RT-PCR)

Total RNA was extracted using TRIzol reagent (Invitrogen) or the RNA Rapid Extraction Kit (EZBioscience) following the manufacturer’s direction. And then, the isolated RNAs were used for reverse transcription with cDNA synthesis kits (Yeasen). SYBR Green Master Mix (Yeasen) was applied to the RT-PCR process. Finally, the Roche 96/384 holes real-time PCR system (Roche) was used to detect the expression of genes. The GAPDH is the internal control.

Cytoplasmic and nuclear isolation was performed with the NORGEN kit (NGB-21000), following the manufacturer’s instructions. The LINC02820 level was detected by RT-PCR. And U6 was used as the nuclear reference and GAPDH was used as the cytoplasmic reference.

All the sequences of the primers used were shown in Supplementary Table 4. The expression quantity was calculated by the 2^–ΔΔCT^ method.

### Cell transfection

siRNA specific to LINC02820 (SiLINC02820) and control scrambled RNA (SiNC) were synthesized by RiboBio (Guangzhou, China), which is used for transient transfection. The CRISPR inhibit (CRISPRi) method was used to construct a sgRNA lentiviral vector to inhibit the expression of LINC02820 at the transcriptional level, and the relevant sequence is listed in Supplementary Table 5. The LINC02820 overexpression vector (OE-LINC02820) was designed by Kidan Bio company (Guangzhou, China), and the empty vector was used as the negative control (Vector).

In the experiment, siRNA was transfected into cells with Lipofectamine 3000 Transfection Reagent (Thermo Fisher), and the transfection concentration was 100 nM. For lentivirus infection, the lentivirus kit (GeneCopoeia) was used according to the instructions.

### Cellular proliferation

The MTS (3-(4,5-dimethylthiazol-2-yl)-5-(3-carboxymethoxyphenyl)-2-(4- sulfophenyl)- 2H-tetrazolium) method and colony formation assays were applied to evaluate cell proliferation.

For the MTS experiment, 1000 cells/per well were cultured in 96-well plates in which MTT reagent was added 20 μl/per well according to the protocol, and absorbance was read at OD490 after incubated at 37 °C for 2 h.

For the colony formation assay, 500 cells/per well were cultured in six-well plates for 2 weeks. Then colonies were fixed, stained, and count.

### Migration, invasion, and wound healing assays

Transwell assays and wound healing assays were applied to explore the migratory and invasive abilities of the ESCC cells. For the wound healing assays, 2 × 10^5^ cells were cultured in 6-well plates. When the cells grew all over the six-well plates, the sterile tips were used to make the wounds. Images of the wounds were captured at 0 h and suitable time (12/24 h/36/48 h, etc.) were captured under the phase-contrast microscope. The Migration Index was calculated as follows:

$${\mathrm{Migration}}\;{\mathrm{Index}} = \% \left( {1 - \frac{{{\mathrm{scratch}}\;{\mathrm{area}}\;{\mathrm{of}}\;12/24/36/48{\mathrm{h}}}}{{{\mathrm{scratch}}\;{\mathrm{area}}\;{\mathrm{of}}\;0{\mathrm{h}}}}} \right)\%$$

For the migration assay, the 1 × 10^5^ cells were suspended in 200 μl serum-free medium and added to the top chamber (Corning). And 800 μl DMEM with 20% FBS was added to the lower chambers. For the invasion assay, the upper chamber membranes were coated with Matrigel (Corning), the treatment method is the same as the migration assay. After 24 or 48 h, the cells in the bottom chamber were fixed with Methanol, stained with 0.1% crystal violet, and then counted under the microscope.

### Western blotting

Cells were lysed with RIPA lysis buffer (Beyotime) and added the protease inhibitor. Next, the concentration of proteins was measured by a BCA Protein Quantification Kit (Yeasen). The standard western blotting protocol was followed. The primary antibodies included antibodies specific for EMT markers, Desmoplakin (Proteintech, # 25318-1-AP), ZEB2 (Proteintech, # 14026-1-AP), N-cadherin (Proteintech, # 22018-1-AP), E-cadherin (Proteintech, # 20874-1-AP), Vimentin (Proteintech, # 10366-1-AP), Slug (Signalway Antibody, #24463), Snail (Cell Signaling Technology, # 3879 T), GAPDH (Proteintech, #10494-1-AP), N-WASP (Cell Signaling Technology, # 4848 T), Phospho-N-WASP (Affinity Biosciences, # AF7032), Cortactin (Proteintech, # 11381-1-AP), SF3B3 (Abcam, # ab209402), IKKα (Cell Signaling Technology, #11930), IKKβ (Cell Signaling Technology, #8943), Phospho-IKKα/β (Cell Signaling Technology, #2697), Phospho-p65 (Cell Signaling Technology, #3033), IκBα (Cell Signaling Technology, #4814), Phospho-IκBα (Cell Signaling Technology, #2859), and p65 (Cell Signaling Technology, #8242) were detected. The antibody dilution ratios were determined according to the protocol. The reference gene was GAPDH.

### Immunofluorescence assays

The 2 × 10^4^ cells were cultured in eight-well glass slides (Millipore). After the cells were adherent, they were fixed with 4% paraformaldehyde. Cells were then incubated with antibodies overnight at 4 °C. Afterward, the cells were stained with secondary antibodies. DAPI (Solarbio) was used to counterstain the nuclei. Final images were taken via fluorescence microscopy.

### RNA-fluorescence in situ hybridization (RNA-FISH)

The probe against LINCO2820 was marked with cy3, which was designed by RiboBio (Guangzhou, China). The 2 × 10^4^ cell samples were placed in 8-well glass slides (Millipore) until they adhered to the slides. All subsequent steps followed the manufacturer’s directions. Finally, confocal microscopy was used to obtain the images.

### Animal experiment

Twenty-six of 3- to 4-week-old male BALB/c nude mice were supplied by Gempharmatech-GD (Guangzhou, China). The Institutional Animal Care and Use Committee at SYSUCC approved all animal experiments in this study.

Twenty-six mice were randomly divided into two groups. The experimental group (*n* = 13) was injected with the K30 cell line in the footpad, which was infected with lentivirus with an overexpression vector (OE-LINC02820). The K30 cell line transfected with an empty vector was the control group (*n* = 13). The 1 × 10^6^ cells in 50 μl DMEM were injected into the footpad of each mouse to generate a primary tumor. After 4 weeks, the popliteal lymph nodes were isolated and collected in RNAlater solution (Beyotime). And Lymph node volume was measured and extracted. Metastasis was assessed by RT-PCR using specific primers for human HPRT, which does not react with the mouse gene [21, 22]. When the CT ≥35, it defined as non-metastasis was, and metastasis was defined as CT <35. The primers were shown in Supplementary Table 4.

### RNA Pull-down and mass spectrometry

LINC02820 and its antisense strand were transcribed in vitro using MEGASCRIPT T7 Kit (Thermo Scientific) and then biotin-labeled with the Pierce RNA 3’-End Desthiobiotinylation Kit (Thermo Scientific). RNA pull-down was performed with Magnetic RNA-Protein Pull-Down Kit (Thermo Scientific) following the manufacturer’s protocol. Eluted proteins were detected by mass spectrometry and Western blot.

### RNA immunoprecipitation (RIP)

RNA immunoprecipitation (RIP) assays were performed using the EZ-Magna RIP RNA-Binding Protein Immunoprecipitation Kit (Merck Millipore). According to the manufacturer’s protocols, the lysis of ESCC cells was incubated with SF3B3 antibody and the wash times, finally, precipitated LINC02820 was detected by RT-PCR.

### RNA-seq

The total RNA of K180 and K30 functional cell lines were extracted using a Trizol reagent kit (Invitrogen, USA) according to the manufacturer’s protocol. RNA quality was assessed on an Agilent 2100 Bioanalyzer (Agilent Technologies, USA) and checked using RNase-free agarose gel electrophoresis. After total RNA was extracted, eukaryotic mRNA was enriched by Oligo(dT) beads. Then the enriched mRNA was fragmented into short fragments using fragmentation buffer and reverse transcript into cDNA with random primers. And sequenced using Illumina HiSeq2500 by GeneDenovo Biotechnology.

### Statistical analysis

GraphPad Prism 8.0 and SPSS 26.0 (IBM) were used for data analyses and visualization. The analysis of correlations between LINC02820 levels and the clinicopathological parameters of ESCC was performed using the *χ*^2^ test (calculated by the Pearson chi-square test or Fisher test). Kaplan–Meier survival analysis was used for survival analysis. Differences between the two groups of data were determined with the Student’s *t*-test. For all analyses, a *p* value <0.05 was considered statistically significant. All data represent the mean ± SD.

## Results

### LINC02820 are upregulated in ESCC and associated with ESCC pathological stage

**Fig. 1:**
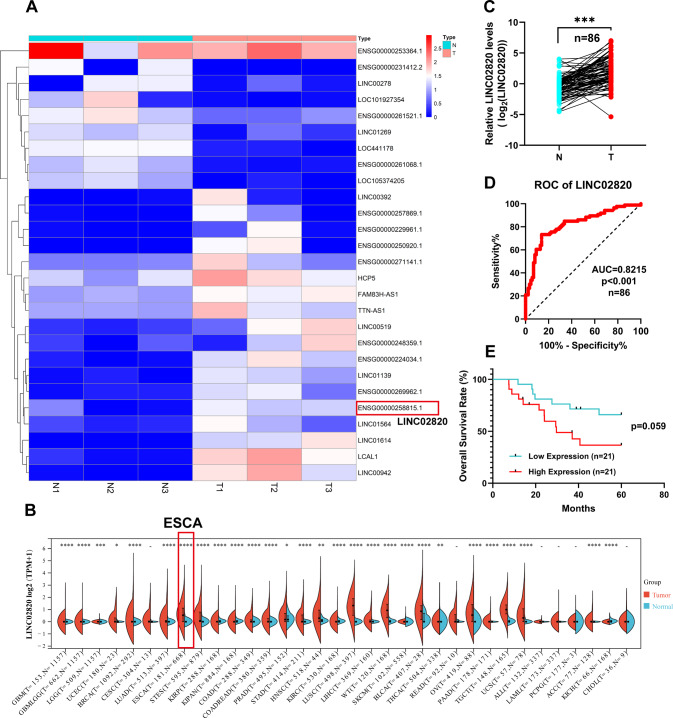
LINC02820 is upregulated in ESCC. **A** Heatmap of 27 differentially expressed lncRNAs in three paired ESCC tissues and adjacent normal tissues. **B** The LINC02820 levels in different cancers. **C** LINC02820 RNA levels were detected by qRT-PCR in 86 pairs of ESCC and adjacent normal tissues. **D** Receiver operating characteristic (ROC) curve analysis of LINC02820 in ESCC (*n* = 86). **E** The overall survival analysis of 42 ESCC patients (*n* = 42, *p* = 0.059). *p < 0.05, **p < 0.01, ***p < 0.001, ****p < 0.0001.

We identified 27 differentially expressed lncRNAs in the three paired ESCC tissues and normal tissues by high-throughput RNA-seq (|log2FC|> 1 and a *p* value <0.05) (Fig. 1A and Supplementary Table 6). We found that some lncRNAs were upregulated in ESCC samples. Subsequently, we focused on the LINC02820, which had no reports on its function in cancers. Our pan-caner analysis of LINC02820 showed that it was increased in many cancers, especially esophageal cancer (Fig. 1B and Supplementary Table 7).

Furthermore, we measured the expression levels of LINC02820 in 86 paired ESCC tumors and normal tissues. The results showed that the LINC02820 levels in ESCC were abnormally increased compared with normal tissues (Fig. 1C, *p* < 0.001). The receiver operating characteristic (ROC) curves of 86 paired samples also implied that LINC02820 can be a biomarker for the ESCC (Fig. 1D, AUC = 0.8215, *p* < 0.001). To further investigate the relationship between LINC02820 levels and the pathological features of ESCC patients, we analyzed the expression of LINC02820 in 86 patients who collected the relevant pathological parameters and divided them into groups. They were then classified according to LINC02820 median expression level. We found that the LINC02820 levels were significantly related to the ESCC pathological stage (Table 1, *p* < 0.001). The analysis between the expression of LINC02820 and patient overall survival suggests that a high LINC02820 expression might link to a poor prognosis, unfortunately, the *p* valve is not significant and could be caused by samples insufficient (Fig. 1. E, *p* = 0.059). These results suggest that LINC02820 may play an important role in ESCC.

**Table 1:** Correlation of LINC02820 levels and clinicopathological parameters in ESCC.

Parameters	Cases(*n* = 86)	LINC02820 expression		*χ*^2^	*p* value
		Low(*n* = 43)	High(*n* = 43)		
**Gender**				0.508	0.476
Male	61	29	32		
Female	25	14	11		
**Age(years)**^a^				1.163	0.281
<61	43	19	24		
≥61	43	24	19		
**Smoking status**				0.047	0.828
No	37	19	18		
Yes	49	24	25		
**Differentiation**				0.871	0.647
Well(G_X_-G1)	12	6	6		
Moderate(G2)	40	22	18		
Poor(G3)	34	15	19		
**pT status**				1.484	0.223
T_is+1+2_	23	9	14		
T_3+4_	63	34	29		
**pN status**				2.299	0.129
N_0_	47	20	27		
N_1-3_	39	23	16		
**pM status**				-	-
M_0_	86	43	43		
M_1_	0	0	0		
**Pathological stage**				9.161	**0.019** ^**b**^
0 + I	7	2	5		
II	44	20	24		
III	31	21	10		
IV	4	0	4		

^a^average age

^b^Fisher test

### LINC02820 doesn’t affect proliferation but promotes migration and invasion of ESCC in vivo and in vitro

**Fig. 2:**
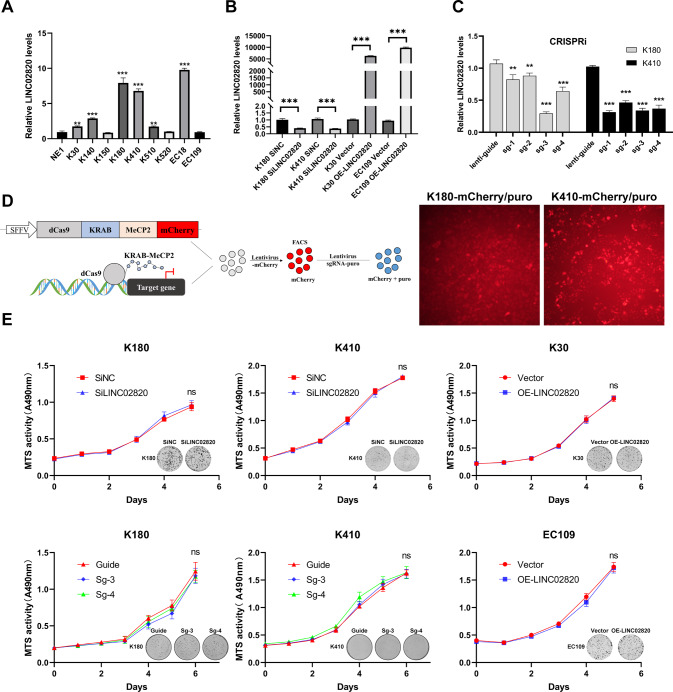
The construction of functional cell lines and LINC02820 does not affect proliferation in ESCC Cells. **A** The expression of LINC02820 in ESCC cell lines. **B** Analysis of LINC02820 in ESCC cells transfected with siRNA(SiLINC02820) or negative control (SiNC), and transfected with overexpression plasmid (OE- LINC02820) or empty vector plasmid (Vector). **C** Analysis of LINC02820 in ESCC cells transfected with LINC02820 suppression plasmid (sg-1, sg-2, sg-3, and sg-4) or control plasmid (lenti-guide or guide). **D** Schematic diagram of CRISPRi/dCas9 and the fluormicrographs of K180/K410 cells transfected with LINC02820 suppression plasmid. **E** The proliferation of K180, K410, K30, and EC109 cells by MTS assay and colony formation experiment. *p < 0.05, **p < 0.01, ***p < 0.001.

To determine the role of LINC02820 in ESCC, we examined its expression in ESCC cell lines by qRT-PCR (Fig. 2A). We found that LINC02820 levels in K180 and K410 cell lines were significantly higher than in the immortalized normal esophageal epithelial cell line (NE1). We then knockdown LINC02820 expression in K180 and K410 cells by transfection of siRNA (SiLINC02820) (Fig. 2B). In addition, the CRISPRi method was used to inhibit the expression of LINC02820 for a long time (Fig. 2C, D). Meanwhile, we found that the copy numbers of LINC02820 in K180 and K410 were decreased after inhibiting the expression of LINC02820 (Supplementary Fig. 1A). Also, we chose the K30 and EC109 cell lines to overexpress (Fig. 2B), which were a low copy of the LINC02820 (Supplementary Fig. 1A).

**Fig. 3:**
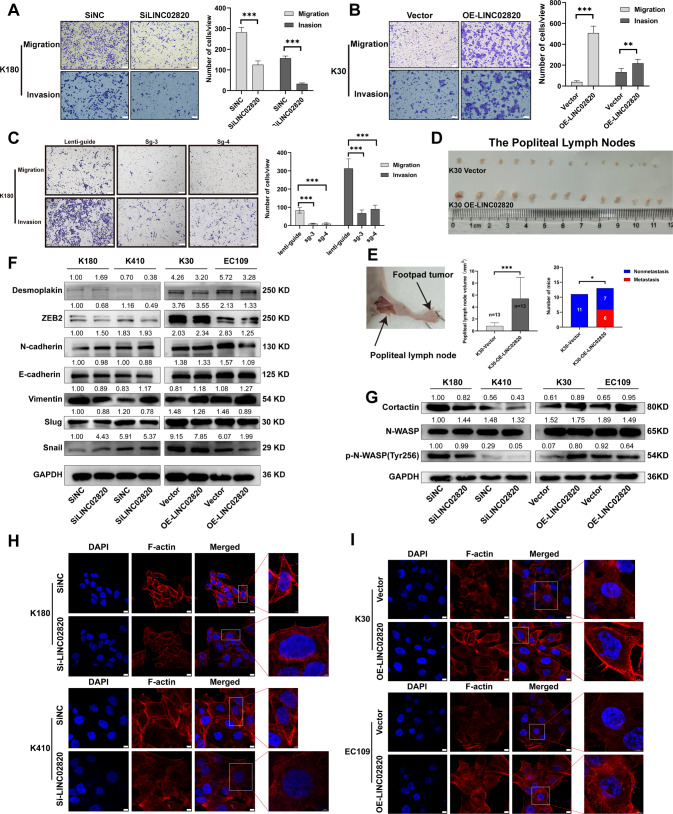
LINC02820 promotes metastasis in ESCC via cytoskeletal remodeling, not EMT. **A** Analysis of the migration and invasion ability of K180 cells transfected with SiLINC02820 or siNC (scale bar, 100 μm). **B** Analysis of the migration and invasion ability of K30 cells transfected with OE- LINC02820 or Vector (scale bar, 100 μm). **C** The migration and invasion ability of K180 cells transfected with LINC02820 suppression plasmid (sg-3 and sg-4) or control plasmid (Guide) (scale bar, 100 μm). **D**, **E** K30 Vector and K30 OE-LINC02820 cell lines were injected in mice footpads and the popliteal lymph node of nude mice was shown, and the metastasis of the popliteal lymph node. **F** The protein levels of EMT markers when LINC02820 are inhibited or increased. **G** The levels of N-WASP, p-N-WASP, and Cortactin about invasive pseudopodia. **H** Inhibition of LINC02820 reduced the F-actin in K180 and K410 cells (scale bar, 10 μm). **I** Overexpression of LINC02820 increased the F-actin in K30 and EC109 cells (scale bar, 10 μm). *p < 0.05, **p < 0.01, ***p < 0.001.

Then, through both MTS and colony formation assays, we found that changing LINC02820 expression did not affect the proliferation of ESCC cell lines (Fig. 2E and Supplementary Fig. 1B–D). However, the migratory and invasion abilities were decreased after LINC02820 was silenced and inversely increased in overexpressed cell lines via Transwell assays (Fig. 3A–C and Supplementary Fig. 1E–G). Similarly, ESCC migratory and invasion abilities declined when we restrained LINC02820 expression in the wound healing assays (Supplementary Fig. 2A-C, G, H). In contrast, ESCC migratory ability increased when LINC02820 was overexpressed (Supplementary Fig. 2D–F).

For in vivo experiments, spontaneous lymph node metastasis experiments were performed to explore ESCC migratory and invasive abilities by injecting an overexpressed K30 cell line into the mouse footpad. We observed that the lymph nodes of OE-LINC02820 mice exhibited more metastasis than the control mice (Fig. 3D, E). In summary, those data suggest that LINC02820 is crucial for the metastasis of ESCC.

### LINC02820 promotes metastasis of ESCC through the cytoskeleton remodeling

To explore how LINC02820 promotes ESCC metastasis, we assessed their epithelial-mesenchymal transition (EMT) progression, as it usually affects migration and invasion abilities. However, we found that the change of LINC02820 expression did not impact numerous EMT markers (Fig. 3F, and Supplementary Fig 3A, B). This indicated that LINC02820 may not work via the EMT pathway. So, we examined the cytoskeleton and invadopodia to explore the possible way, which is instrumental for cancer cell migration [23, 24]. We firstly examined markers for invadopodia, including Cortactin, N-WASP, and phosphorylated N-WASP (p-N-WASP), and discovered that the protein levels of Cortactin and p-N-WASP were decreased when LINC02820 was silenced in K180 and K410 cells (Fig. 3G). The phenomenon was the opposite in K30 and EC109 (Fig. 3G). This prompted us to investigate whether LINC02820 can induce F-actin malfunction by transforming it into G-actin. We found that LINC02820 was decreased in K180 and K410 cell lines, F-actin levels decreased and cellular morphology changed (Fig. 3H). Whereas, F-actin increased after LINC02820 overexpression in K30 and EC109 cell lines (Fig. 3I). These data show that cytoskeleton remodeling is also an important factor affecting metastasis in ESCC.

### LINC02820 is mainly distributed in the nucleus and might be involved in the TNF/NF-κB signaling pathway

**Fig. 4:**
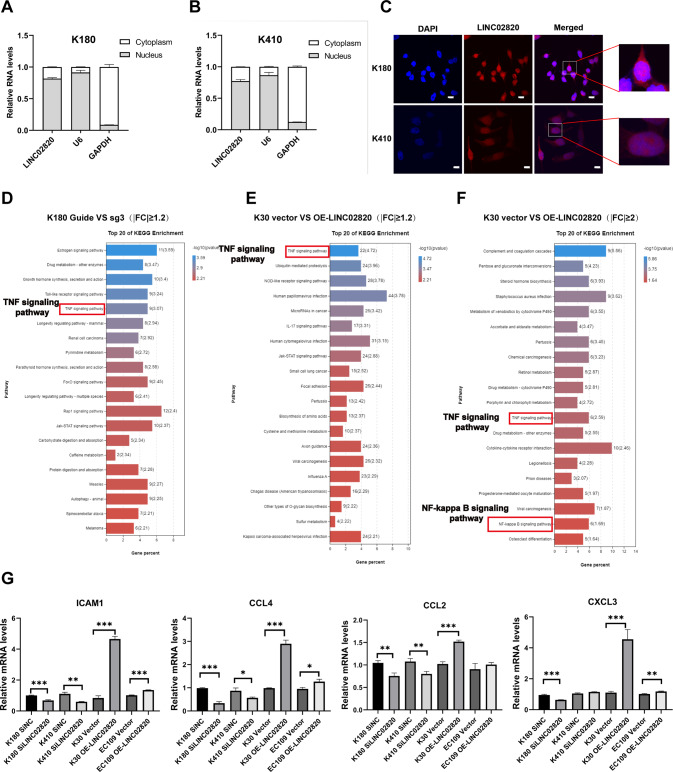
LINC02820 is mainly distributed in the nucleus and involved in TNF/NF-κB signaling pathways. **A**, **B** Subcellular separation experiment for K180 and K410 cells. GAPDH (cytoplasmic control), U6 (nuclear control). **C** RNA-FISH was performed to determine LINC02820 location in ESCC cells (scale bar, 10 μm). **D** The top 20 KEGG enrichment pathway analyses between K180 cells transfected with SiLINC02820 or SiNC based on RNA-seq (|FC|≥1.2). **E** The top 20 KEGG enrichment pathway analyses between K30 cells transfected with vector or OE-LINC02820 based on RNA-seq (|FC|≥1.2). **F** The top 20 KEGG enrichment pathway analyses between K30 cells transfected with vector or OE-LINC02820 based on RNA-seq (|FC|≥2.0). **G** Downstream genes expression of NF-κB signaling pathway. *p < 0.05, **p < 0.01, ***p < 0.001.

It has been reported that the function of lncRNA relies on its subcellular localization [9]. To further discern LINC02820’s mechanism, we conducted a positioning test LINC02820. We demonstrated that LINC02820 is enriched in the nucleus of K180 and K410 cells (Fig. 4A–C). In addition, the nuclear LINC02820 has been inhibited when LINC02820 is downregulated in K180 and K410 cells (Supplementary Fig. 4A), on the contrary, nuclear LINC02820 has been augmented in K30 cells transfected with OE- LINC02820 (Supplementary Fig. 4A). what’s more, we used lncATLAS (https://lncatlas.crg.eu/) to predict the subcellular location of LINC02820 and found it may be located in the nucleus (Supplementary Fig. 4B).

Next, we aimed to explore the potential molecular mechanisms of LINC02820 in ESCC. KEGG analysis of two pairs of matched cells indicated that the TNF signaling pathway (screened by |FC|)≥1.2 and *p* value <0.05) in ESCC was involved in tumorigenesis (Fig. 4D, E). In a previous study, it has been verified that the TNF signaling and NF-κB signaling pathways are correlated [20]. Fortunately, we also found that the TNF signaling and NF-κB signaling pathways were enriched in the K30 cells (screened by |FC|)≥2.0 and *p* value <0.05), indicating that LINC02820 may regulate these pathways (Fig. 4F). Moreover, when LINC02820 was altered, we found the downstream NF-κB signaling pathway factors, such as ICAM1, CCL2, CCL4, and CXCL3, also changed, further indicating that LINC02820 may function by TNF/NF-κB signaling pathway (Fig. 4G).

**Fig. 5:**
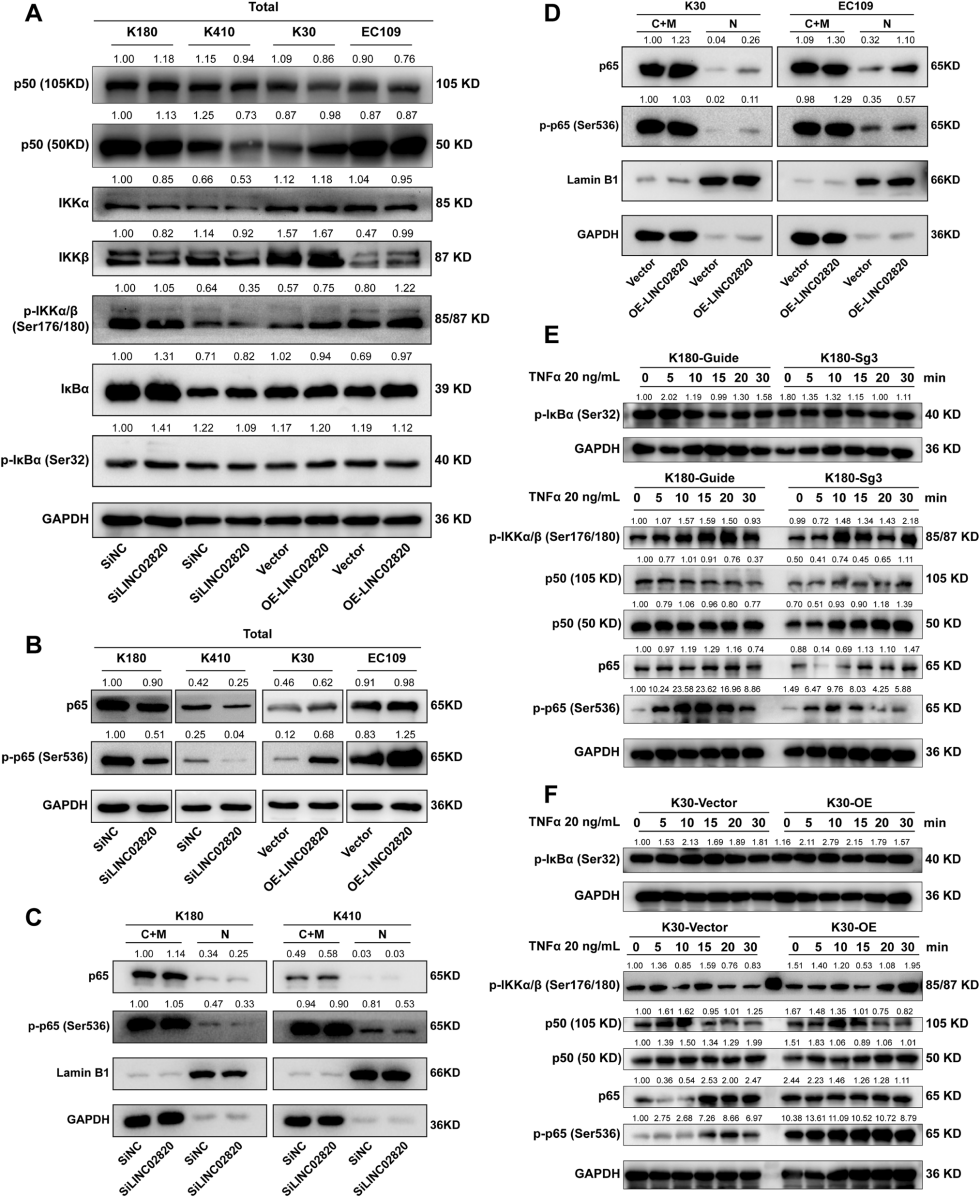
LINC02820 is involved in the TNF/NF-κB signaling pathway. **A** The expression of upstream genes of the NF-κB signal pathway. **B** The changes of LINC02820 affected the expression of p65 and its phosphorylation. **C**, **D** Nuclear and cytoplasmic protein fractions were isolated from ESCC cell lines. **E**, **F** The expression of p65 and p-p65 in K180 and K30 cells in response to TNFα (20 ng/mL).

Furthermore, we found that the change of the LINC02820 makes no sense to the upstream NF-κB signaling pathway of IKKα, IκBα and its phosphorylation (Fig. 5A), but may affect IKKβ and its phosphorylation, though the effect is small (Fig. 5A). However, the change of LINC02820 impacts p65 and its phosphorylation (p-p65) changed, obviously (Fig. 5B). We found that LINC02820 inhibition reduced p65 and p-p65 in ESCC cells (Fig. 5B). In contrast, when LINC02820 was upregulated in K30 and EC109 cells, p65 and p-p65 expression increased (Fig. 5B). And the cytosolic protein and nuclear protein were also separated to detect p-p65 in the nucleus, we found that when LINC02820 was knocked down, nucleic p-p65 was decreased (Fig. 5C) whereas LINC02820 overexpression resulted in an increased nucleic p-p65 (Fig. 5D). Altogether, these data imply that LINC02820 may work in the TNF/NF-κB signaling pathway.

### LINC02820 cooperates with TNFα to amplify the NF-κB signaling pathway and reconstruct the cytoskeleton

**Fig. 6:**
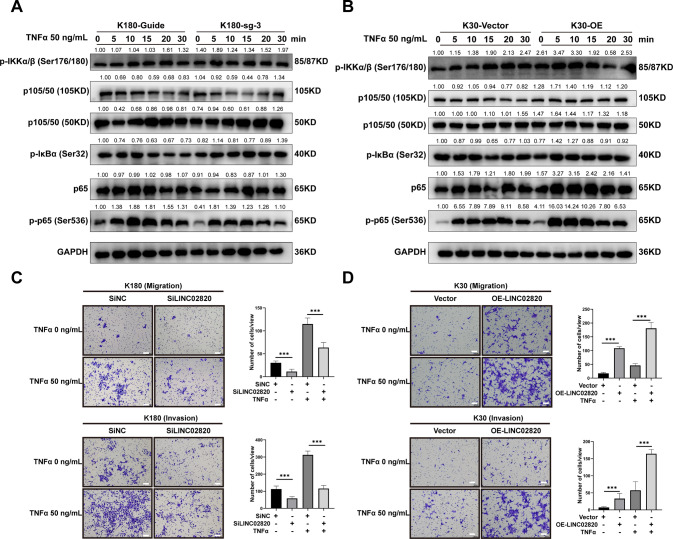
LINC02820 cooperates with TNFα to amplify the NF-κB Signaling Pathway. **A**, **B** The expression of p65 and p-p65 in K180 and K30 cells in response to TNFα (50 ng/mL). **C** The migration and invasion of K180 cells transfected with SiLINC02820 or siNC in response to TNFα (scale bar, 100 μm). **D** The migration and invasion of K30 cells transfected with OE- LINC02820 or Vector in response to TNFα (scale bar, 100 μm).

To further analyze the relationship between LINC02820 and TNF/NF-κB signaling pathways, particularly with migration and invasion capabilities, we used tumor necrosis factor-alpha (TNFα) to stimulate K30 and K180. In the assays, we used low and high concentrations of TNFα (20 or 50 ng/mL) to stimulate ESCC cell lines at different time points (0, 5, 10, 15, 20, and 30 min). We found that the expression of p-IKKα, p65, and p-p65 were altered (Figs. 5E, F, 6A, B). By looking at the cytosolic and nuclear protein of p65 and p-p65, we found that LINC02820 cooperates with TNFα to promote the nuclear translocation of p65, thus regulating the NF-κB signaling pathway (Supplementary Fig. 5A–D).

And we also found that the migration and invasion abilities of K180 with lower LINC02820 expression exhibited partial recovery when TNFα was added (Fig. 6C). What’s more, K30 cells with overexpressed LINC02820 exhibited significantly improved abilities after TNFα was added (Fig. 6D).

**Fig. 7:**
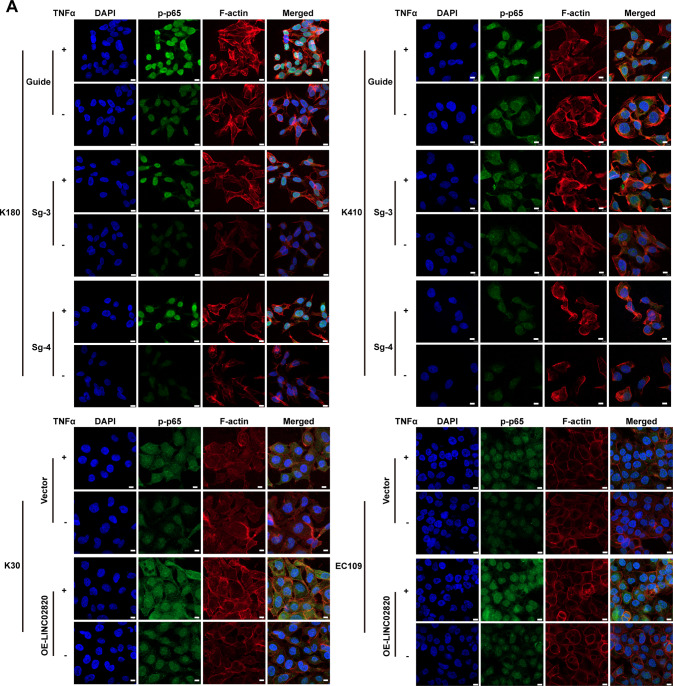
LINC02820 cooperated with TNFα to promote p-p65 into the nucleus and reconstruct the cytoskeleton. **A** The expression of p-p65 and F-actin in K180, K410, K30, and EC109 cells under TNFα (20 ng/mL) (scale bar, 10 μm).

In addition, immunofluorescence assays of p-p65 and F-actin visually demonstrate that the level of p-p65 entering the nucleus was increased when LINC02820 was reduced while TNFα was added, notably, the level of p-p65 entering the nucleus was still lower than that of the control group (Fig. 7A). Moreover, when LINC02820 was overexpressed and TNFα was added in K30 and EC109, p-p65 entering the nucleus was increased and the cytoskeleton remodeled (Fig. 7A).

Therefore, we believe LINC02820 synergies with TNFα to magnify the NF-κB signaling pathway and promote cytoskeletal remodeling, thus affecting ESCC metastasis.

### LINC02820 may interact directly with SF3B3 to participate in the NF-κB signaling pathway through alternative splicing

**Fig. 8:**
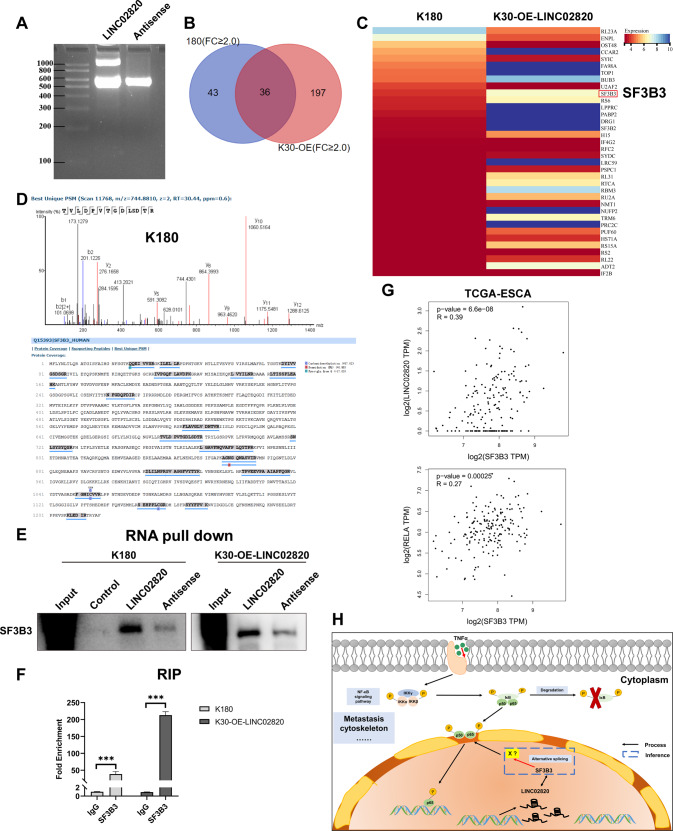
LINC02820 interacts directly with SF3B3 in ESCC. **A** The integrity of RNA probes was detected by formaldehyde denaturing gel electrophoresis. **B**, **C** Eluates were analyzed by liquid chromatography-tandem mass spectrometry (LC-MS/MS) to identify immunoprecipitated proteins. **D** Analysis of SF3B3 in K180 cells by liquid chromatography-tandem mass spectrometry (LC-MS/MS). **E** The binding between LINC02820 and SF3B3 was confirmed by western blot. **F** The result of RNA immunoprecipitation. **G** The correlation analysis between LINC02820 and SF3B3 or SF3B3 and p65(RELA). **H** Proposed model for LINC02820 in ESCC. *p < 0.05, **p < 0.01, ***p < 0.001.

To further clarify how LINC02820 collaborates with TNFα to promote ESCC metastasis, RNA pull-down combined with mass spectrometry was performed. We prepared LINC02820 probes and the antisense strand as a control (Fig. 8A) and incubated them with whole-cell lysates of endogenous high LINC02820 expression K180 cells and exogenous high LINC02820 expression K30 cells. Mass spectrometric indicated that LINC02820 may interact with SF3B3 (Fig. 8B–D and Supplementary Fig. 5E), which was further confirmed with western blot (Fig. 8E). RIP assays showed that SF3B3 can directly interact with LINC02820 in ESCC cells (Fig. 8F).

Furthermore, the correlation analysis in ESCA showed that LINC02820 expression was positively correlated with SF3B3 expression and SF3B3 expression positively correlated with p65(RELA) expression (Fig. 8G). Considering SF3B3 is a subunit of U2, a small nuclear ribosomal protein, and is involved in the alternative splicing of RNA. Taken together, we conclude that LINC02820 may function by interacting directly with SF3B3 and contribute to metastasis in ESCC by regulating the NF-κB signaling pathway (Fig. 8H).

## Discussion

ESCC is a global oncology problem and metastasis is one of the main reasons leading to poor prognosis of ESCC patients. Understanding the metastatic molecular mechanism of ESCC will provide more treatment strategies for ESCC patients and improve the prognosis of patients with ESCC. In recent years, several studies have shown that lncRNA can participate in ESCC metastasis. For example, lncRNA VESTAR can promote lymph node metastasis in ESCC [25], and lncRNA CASC9 upregulates the expression of LAMC2 to promote metastasis [26]. And lncRNA HOTTIP can regulate ESCC metastasis at both transcriptional and post-transcriptional levels [27]. Those disease-associated lncRNA signatures may be useful for developing novel biomarkers and therapeutic targets for cancers, especially with the development of delivery systems for RNA therapy, like polymer-based, lipid-based, and conjugate-based drug delivery systems [28]. In our study, we found the F-actin had remodeled with the change of LINC02820 and may form “invadopodia” on the cell surface. The cytoskeleton is the driving force of cell movement, which are crucial for tumor cell metastasis [29, 30]. Therefore, we believe that LINC02820 is crucial for ESCC.

Meanwhile, many reports are indicating that lncRNAs impact the metastasis of ESCC through the NF-κB signaling pathway. For example, lncRNA NKILA can inhibit the metastasis of ESCC by the NF-κB signaling pathway [31], and lncRNA FTH1P3 regulated metastasis and invasion of ESCC through SP1/NF-κB pathway [32]. Moreover, lncRNA FMR1-AS1 upregulates the level of c-MYC and activates the NF-κB signaling pathway to promote the invasion of ESCC [33]. All data show that lncRNA and NF-κB signaling pathways can function in ESCC. In the present study, we also found that LINC02820 magnifies the NF-κB signaling pathway by promoting p65 translocation into the nucleus under TNFα stimulation. And through the RNA pull-down-MS experiment, we have found that LINC02820 interacts with SF3B3, which is a part of the spliceosome and participates in the precursor-mRNA (pre-mRNA) splicing reaction [34]. It is reported that SF3B3 is a key regulator of pre-mRNA splicing of EZH2, therefore regulating tumor development [35, 36]. Thus, we speculated that LINC02820 might interact with SF3B3 to influence the alternative splicing of pre-mRNA for some specific genes (we called “X”) and further activate the NF-κB signaling pathway to promote the metastasis in ESCC.

However, we are still left with some questions. We still don’t know what caused the dysregulation of LINC02820. There are many reasons for it, such as gene copy number change [37], DNA methylation alteration [38], and transcript stability [39]. And we do not fully understand the specific molecular mechanism of LINC02820 interacting with SF3B3 to affect the pre-mRNA alternative splicing and impact the NF-κB signaling pathway. These all need more experiments to prove.

In summary, our study found a novel lncRNA, LINC02820, which is upregulated in ESCC and impacts metastasis of ESCC via cytoskeletal remodeling. According to a series of assays, we found that LINC02820 may bind to SF3B3 to function, and can cooperate with TNFα to regulate the NF-κB signaling pathway to work in ESCC (Fig. 8H). All in all, our findings contributed to revealing the molecular mechanism of ESCC and revealed that LINC02820 may be a new target for the diagnosis and treatment of ESCC.

## Supplementary information

Supplementary Table 1

Supplementary Table 2

Supplementary Table 3

Supplementary Table 4

Supplementary Table 5

Supplementary Table 6

Supplementary Table 7

supplementary figure-1

supplementary figure-2

supplementary figure-3

supplementary figure-4

supplementary figure-5

Supplementary Figures and Table Legends

## Data Availability

Raw data from this study have been deposited to the Research Data Deposit database (www.researchdata.org.cn) under accession number RDDB2022788640.
