# Supplementary material for: Novel long noncoding RNA LINC02820 augments TNF signaling pathway to remodel cytoskeleton and potentiate metastasis in esophageal squamous cell carcinoma
Source: Cancer Gene Ther. 2022 Nov 10;30(2):375–87. doi: 10.1038/s41417-022-00554-2 (PMC9935391; doi:10.1038/s41417-022-00554-2)
Supplement: Supplementary file 1 — Supplementary Table 1 [file 41417_2022_554_MOESM1_ESM.docx]

**Supplementary Table 1.**

**The Information of LINC02820 in Different Databases**

| Database | Name/ID | URL |
| --- | --- | --- |
| Ensembl | ENSG00000258815.1 | <http://asia.ensembl.org/Homo_sapiens/Gene/Summary?db=core;g=ENSG00000258815;r=12:85318060-85342912;t=ENST00000555596> |
| Ensembl/GENCODE | ENST00000555596.1 | <https://asia.ensembl.org/Homo_sapiens/Transcript/Summary?db=core;g=ENSG00000258815;r=12:85318060-85342912;t=ENST00000555596> |
| NONCODE | NONHSAT029748.2 | [http://www.noncode.org/show_rna.php?id=NONHSAT029748&version=2&utd=1#](http://www.noncode.org/show_rna.php?id=NONHSAT029748&version=2&utd=1) |
| LNCipedia | lnc-ALX1-2:5 | <https://lncipedia.org./db/transcript/lnc-ALX1-2:5> |
| MalaCards | LINC02820 | <https://www.malacards.org/search/results/LINC02820> |
| GeneCards | LINC02820 | <https://www.genecards.org/cgi-bin/carddisp.pl?gene=LINC02820> |
| HGNC | 54351 | <https://www.genenames.org/data/gene-symbol-report/#!/hgnc_id/HGNC:54351> |

**The Sequence of LINC02820：**

5’-AGACTGCTGTGCTAGCAATCAGCGAGACTCCGTGGGCGTAGGACCCTCCGAGCCAGGCATCAGCTATCAAAACTTATGGGAAATGGAATTGAAGACAACTACTTCTAGAATTGGTGCATGGAAGCTTCTCATAGGTGATTAGCCACACTCTTCTTCTGTCAACCTGGTGCAGAAAAGCTTGAAGACCTTGGAAATCACATATTGATGATGGAGGAGATACAAGAATGAAGGACCCTGGGTTCCTGAATCAACACTCTAAGAAGAGACTCCTTGAAATCAAGAGCACTCATTTTGACATTGCAAACGGAAAAATTCACTTCTATCAGGTTTGAACCAATATATAGTTTAGGGTTTTTAAAAAATAACTGTTAGCATGACTTTAACTAATACACATGCCATACAGGATCAGACCTCTGTGACTTTGCTTTTACAGTCTGCTCTGTCTGGAGGGGAGTTTCATCTTTGCCATCCTTAATGATATAGCATAGACGCTATGTCCTCCAGAAACTTCCTTGGCAC-3'
