# Supplementary material for: Novel long noncoding RNA LINC02820 augments TNF signaling pathway to remodel cytoskeleton and potentiate metastasis in esophageal squamous cell carcinoma
Source: Cancer Gene Ther. 2022 Nov 10;30(2):375–87. doi: 10.1038/s41417-022-00554-2 (PMC9935391; doi:10.1038/s41417-022-00554-2)
Supplement: Supplementary file 3 — Supplementary Table 3 [file 41417_2022_554_MOESM3_ESM.docx]

**Supplementary Table 3.**

**The Information of ESCC Cell Lines**

| **Cell lines** | **Disease** | **Species** | **Age/Sex** | **Differentiation** | **URL** |
| --- | --- | --- | --- | --- | --- |
| NE1 | Immortalized normal esophageal  epithelial cell line | Homo sapiens | 72/M | A human papillomavirus  E6/E7/telomerase–  immortalized normal  esophageal epithelial cell line | <https://web.expasy.org/cellosaurus/CVCL_E306> |
| KYSE30 | Esophageal squamous cell carcinoma | Homo sapiens | 64/M | WD | <https://web.expasy.org/cellosaurus/CVCL_1351> |
| KYSE140 | Esophageal squamous cell carcinoma | Homo sapiens | 54/M | MD | <https://web.expasy.org/cellosaurus/CVCL_1347> |
| KYSE150 | Esophageal squamous cell carcinoma | Homo sapiens | 49/F | PD | <https://web.expasy.org/cellosaurus/CVCL_1348> |
| KYSE180 | Esophageal squamous cell carcinoma | Homo sapiens | 53/M | WD | <https://web.expasy.org/cellosaurus/CVCL_1349> |
| KYSE410 | Esophageal squamous cell carcinoma | Homo sapiens | 51/M | PD | <https://web.expasy.org/cellosaurus/CVCL_1352> |
| KYSE510 | Esophageal squamous cell carcinoma | Homo sapiens | 67/F | WD | <https://web.expasy.org/cellosaurus/CVCL_1354> |
| KYSE520 | Esophageal squamous cell carcinoma | Homo sapiens | 58/F | MD | <https://web.expasy.org/cellosaurus/CVCL_1355> |
| EC18 | Esophageal squamous cell carcinoma | Homo sapiens | Unknown/M | PD | <https://web.expasy.org/cellosaurus/CVCL_RX98> |
| EC109 | Esophageal squamous cell carcinoma | Homo sapiens | 37/F | MD | <https://web.expasy.org/cellosaurus/CVCL_6898> |

PD: Poorly differentiated;

MD: Moderate differentiated;

WD: Well differentiated.
