# Supplementary material for: Novel long noncoding RNA LINC02820 augments TNF signaling pathway to remodel cytoskeleton and potentiate metastasis in esophageal squamous cell carcinoma
Source: Cancer Gene Ther. 2022 Nov 10;30(2):375–87. doi: 10.1038/s41417-022-00554-2 (PMC9935391; doi:10.1038/s41417-022-00554-2)
Supplement: Supplementary file 4 — Supplementary Table 4 [file 41417_2022_554_MOESM4_ESM.docx]

**Supplementary table 4.**

**The primers for RT-PCR**

| Name | Sequence (5’-3’) |
| --- | --- |
| LINC02820-Forward | 5’-AGCTTCTCATAGGTGATTAGCCA-3’ |
| LINC02820-Reverse | 5’-GTGTTGATTCAGGAACCCAGG-3’ |
| GAPDH-Forward | 5’-GTCTCCTCTGACTTCAACAGCG-3’ |
| GAPDH -Reverse | 5’-ACCACCCTGTTGCTGTAGCCAA-3’ |
| U6-Forward | 5’-CAGCACATATACTAAAATTGGAACG-3’ |
| U6-Reverse | 5’-ACGAATTTGCGTGTCATCC-3’ |
| ICAM-Forward | 5’-AGCGGCTGACGTGTGCAGTAAT-3’ |
| ICAM-Reverse | 5’-TCTGAGACCTCTGGCTTCGTCA-3’ |
| CCL4-Forward | 5’-GCTTCCTCGCAACTTTGTGGTAG-3’ |
| CCL4-Reverse | 5’-GGTCATACACGTACTCCTGGAC-3’ |
| CCL2-Forward | 5’-AGAATCACCAGCAGCAAGTGTCC-3’ |
| CCL2-Reverse | 5’-TCCTGAACCCACTTCTGCTTGG-3’ |
| CXCL3-Forward | 5’-TTCACCTCAAGAACATCCAAAGTG-3’ |
| CXCL3-Reverse | 5’-TTCTTCCCATTCTTGAGTGTGGC-3’ |
| HPRT-Forward | 5’-TTCCTTGGTCAGGCAGTATAATCC-3’ |
| HPRT-Reverse | 5’-AGTCTGGCTTATATCCAACACTTCG-3’ |
| ACTB-Forward | 5’-CAATGAGCTG CGTGTGGC-3’ |
| ACTB-Reverse | 5’-CGTACATGGCTGGGGTGTT-3’ |
