# Supplementary material for: Novel long noncoding RNA LINC02820 augments TNF signaling pathway to remodel cytoskeleton and potentiate metastasis in esophageal squamous cell carcinoma
Source: Cancer Gene Ther. 2022 Nov 10;30(2):375–87. doi: 10.1038/s41417-022-00554-2 (PMC9935391; doi:10.1038/s41417-022-00554-2)
Supplement: Supplementary file 5 — Supplementary Table 5 [file 41417_2022_554_MOESM5_ESM.docx]

**Supplementary table 5.**

**The sequence of making Smart silencer RNA and sgRNA plasmids**

| Name | Sequence (5’-3’) |
| --- | --- |
| SiRNA | 5’-CCTGAATCAACACTCTAAGA-3’  5’-CAACCTGGTGCAGAAAAGCT-3’  5’-TGACTTTAACTAATACACAT-3’  5’-GGAATTGAAGACAACTACT-3’  5’-CTGGGTTCCTGAATCAACA-3’  5’-GTTAGCATGACTTTAACTA-3’ |
| sg-1-F | 5’-CACCGAGGTGCGTGATATAATCTCG-3’ |
| sg-1-R | 5’-AAACCGAGATTATATCACGCACCTC-3’ |
| sg-2-F | 5’-CACCGCAAGGCGGAAGACAGGCTGG-3’ |
| sg-2-R | 5’-AAACCCAGCCTGTCTTCCGCCTTGC-3’ |
| sg-3-F | 5’-CACCGACCCTGAGCGAGCCGAAGCA-3’ |
| sg-3-R | 5’-AAACTGCTTCGGCTCGCTCAGGGTC-3’ |
| sg-4-F | 5’-CACCGAGCCCACCACAGCTCAAGG-3’ |
| sg-4-R | 5’-AAACCCTTGAGCTGTGGTGGGCTC-3’ |
