# Supplementary material for: Novel long noncoding RNA LINC02820 augments TNF signaling pathway to remodel cytoskeleton and potentiate metastasis in esophageal squamous cell carcinoma
Source: Cancer Gene Ther. 2022 Nov 10;30(2):375–87. doi: 10.1038/s41417-022-00554-2 (PMC9935391; doi:10.1038/s41417-022-00554-2)
Supplement: Supplementary file 7 — Supplementary Table 7 [file 41417_2022_554_MOESM7_ESM.docx]

**Supplementary Table 7.**

**The pan-cancer analysis of LINC02820**

| LncRNA is up-regulated in tumors | | | |
| --- | --- | --- | --- |
|  | Tumor (mean±SD) | Normal (mean±SD) | *P*-value |
| GBM | 0.04±0.25 | 1.8e-3±0.02 | 4.3e-9 |
| GBMLGG | 0.01±0.12 | 1.8e-3±0.02 | 6.8e-7 |
| LGG | 4.6e-3±0.03 | 1.8e-3±0.02 | 1.1e-4 |
| UCEC | 0.26±0.47 | 0.02±0.05 | 0.01 |
| BRCA | 0.18±0.47 | 0.02±0.08 | 2.2e-8 |
| LUAD | 0.24±0.56 | 0.02±0.07 | 7.6e-14 |
| **ESCA** | **0.69±0.70** | **0.04±0.11** | **4.9e-60** |
| STES | 0.42±0.58 | 0.03±0.10 | 3.9e-71 |
| COAD | 0.18±0.36 | 0.02±0.10 | 8.3e-14 |
| COADREAD | 0.22±0.41 | 0.02±0.09 | 1.2e-16 |
| PRAD | 0.43±0.55 | 0.27±0.33 | 0.03 |
| STAD | 0.31±0.48 | 9.0e-3±0.05 | 6.6e-25 |
| HNSC | 0.55±0.66 | 0.13±0.16 | 1.6e-3 |
| LUSC | 1.24±0.84 | 0.02±0.07 | 7.9e-116 |
| LIHC | 0.16±0.34 | 0.01±0.04 | 4.3e-9 |
| WT | 0.95±0.71 | 0.06±0.09 | 4.1e-28 |
| SKCM | 0.02±0.07 | 5.0e-3±0.04 | 1.8e-8 |
| BLCA | 0.89±0.79 | 0.30±0.39 | 1.5e-5 |
| OV | 0.79±0.84 | 0.05±0.24 | 3.3e-21 |
| PAAD | 0.06±0.19 | 3.4e-3±0.02 | 4.7e-7 |
| TGCT | 1.04±0.60 | 0.06±0.12 | 5.0e-48 |
| UCS | 0.64±0.80 | 0.03±0.08 | 9.0e-13 |
| ACC | 0.09±0.29 | 1.2e-3±0.01 | 2.6e-5 |
| LncRNA is down-regulated in tumors | | | |
| KIRP | 0.06±0.27 | 0.06±0.09 | 3.4e-19 |
| KIPAN | 0.04±0.21 | 0.06±0.09 | 2.2e-32 |
| KIRC | 0.04±0.19 | 0.06±0.09 | 1.1e-22 |
| THCA | 0.04±0.22 | 0.05±0.17 | 1.9e-3 |
| KICH | 8.8e-3±0.06 | 0.06±0.09 | 1.0e-8 |
| LncRNA is no means in tumors | | | |
| CESC | 0.19±0.42 | 0.01±0.04 | 0.12 |
| READ | 0.32±0.52 | 0.04±0.06 | 0.23 |
| ALL | 9.1e-3±0.05 | 1.7e-3±0.01 | 0.13 |
| LAML | 0.02±0.13 | 1.7e-3±0.01 | 0.81 |
| PCPG | 0.02±0.16 | 0.00±0.00 | 0.78 |
| CHOL | 0.09±0.34 | 0.00±0.00 | 0.31 |
