# Supplementary figures and images for: Novel long noncoding RNA LINC02820 augments TNF signaling pathway to remodel cytoskeleton and potentiate metastasis in esophageal squamous cell carcinoma
Source: Cancer Gene Ther. 2022 Nov 10;30(2):375–87. doi: 10.1038/s41417-022-00554-2 (PMC9935391; doi:10.1038/s41417-022-00554-2)

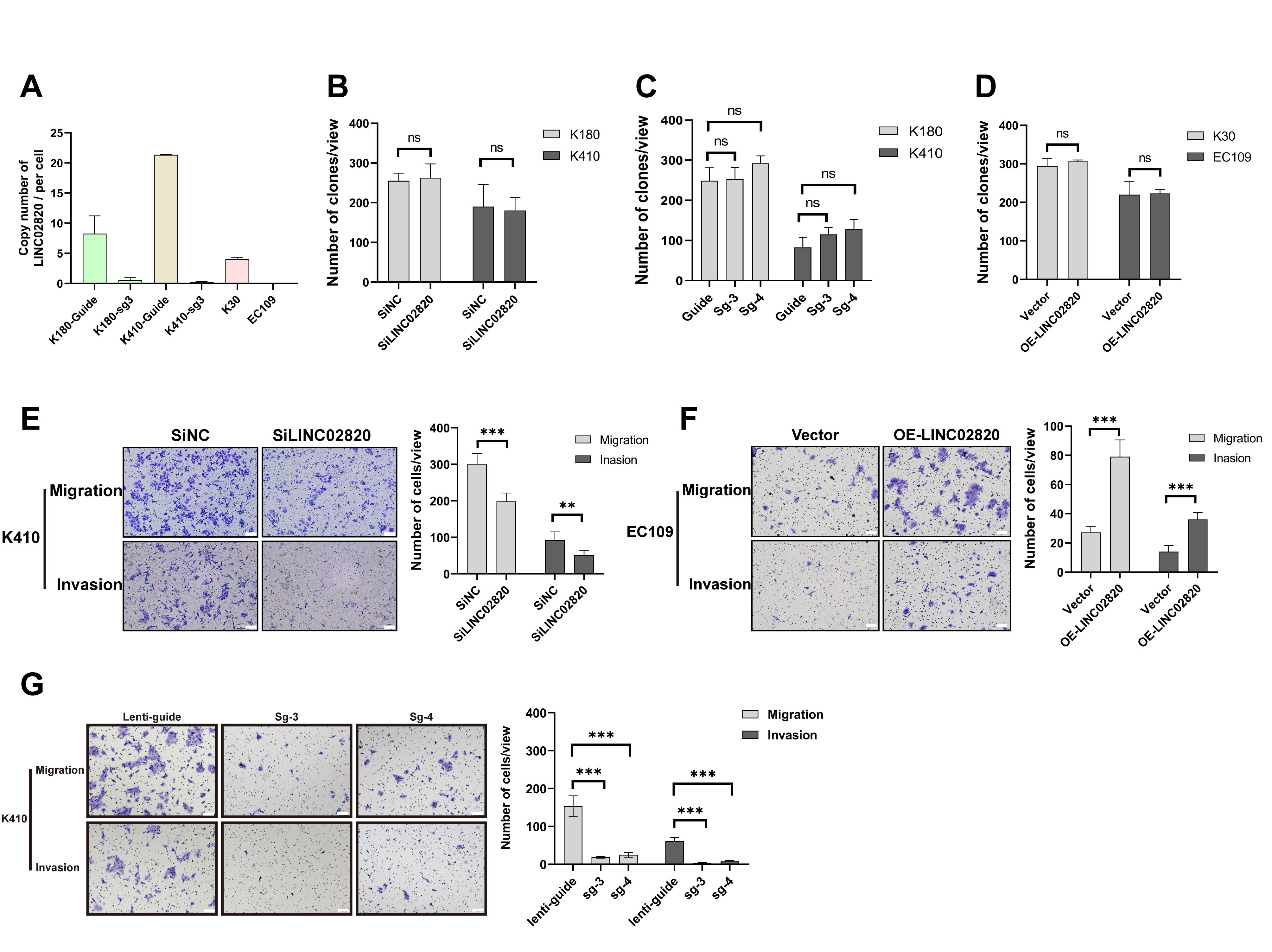

Supplement: Supplementary file 8 — supplementary figure-1 [file 41417_2022_554_MOESM8_ESM.tif]

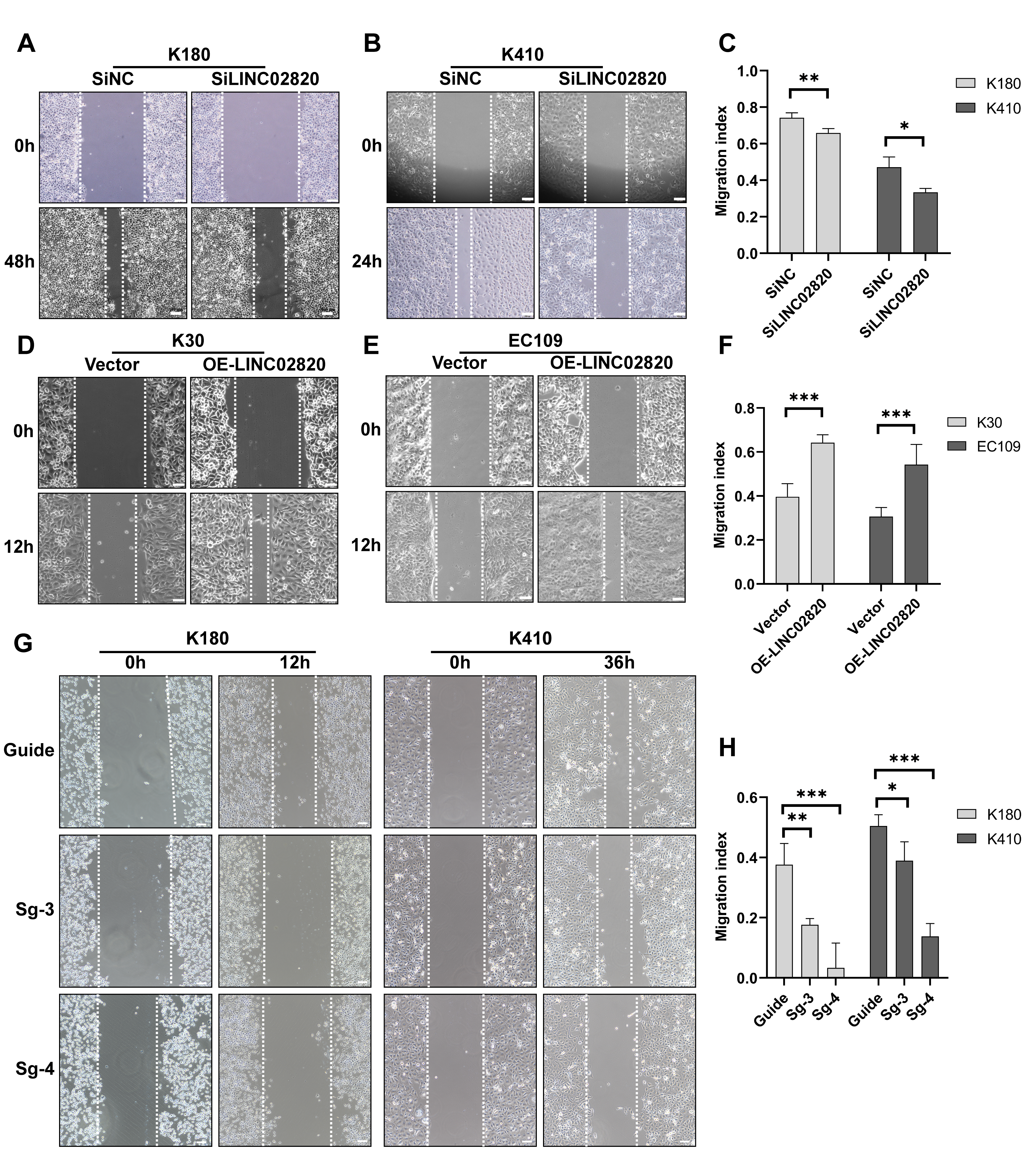

Supplement: Supplementary file 9 — supplementary figure-2 [file 41417_2022_554_MOESM9_ESM.tif]

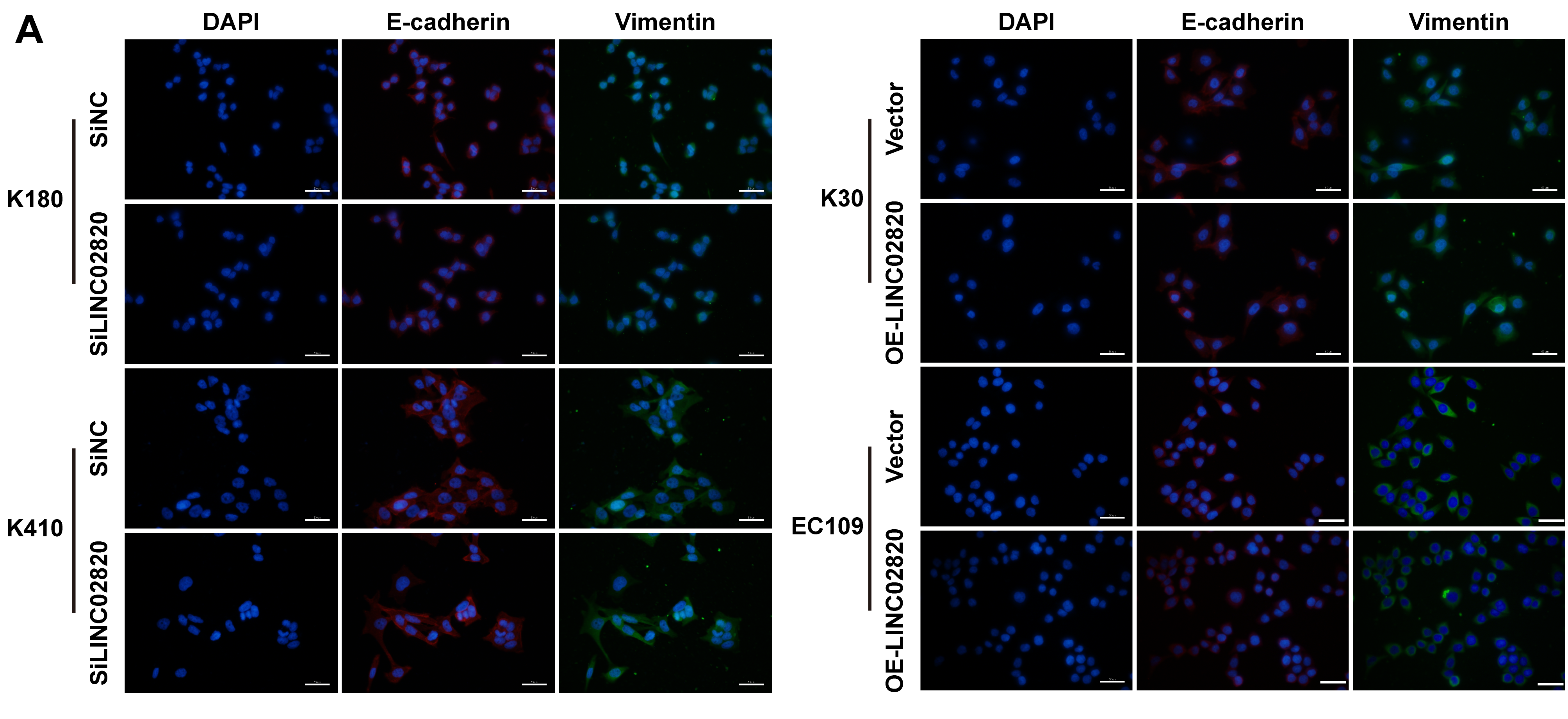

Supplement: Supplementary file 10 — supplementary figure-3 [file 41417_2022_554_MOESM10_ESM.tif]

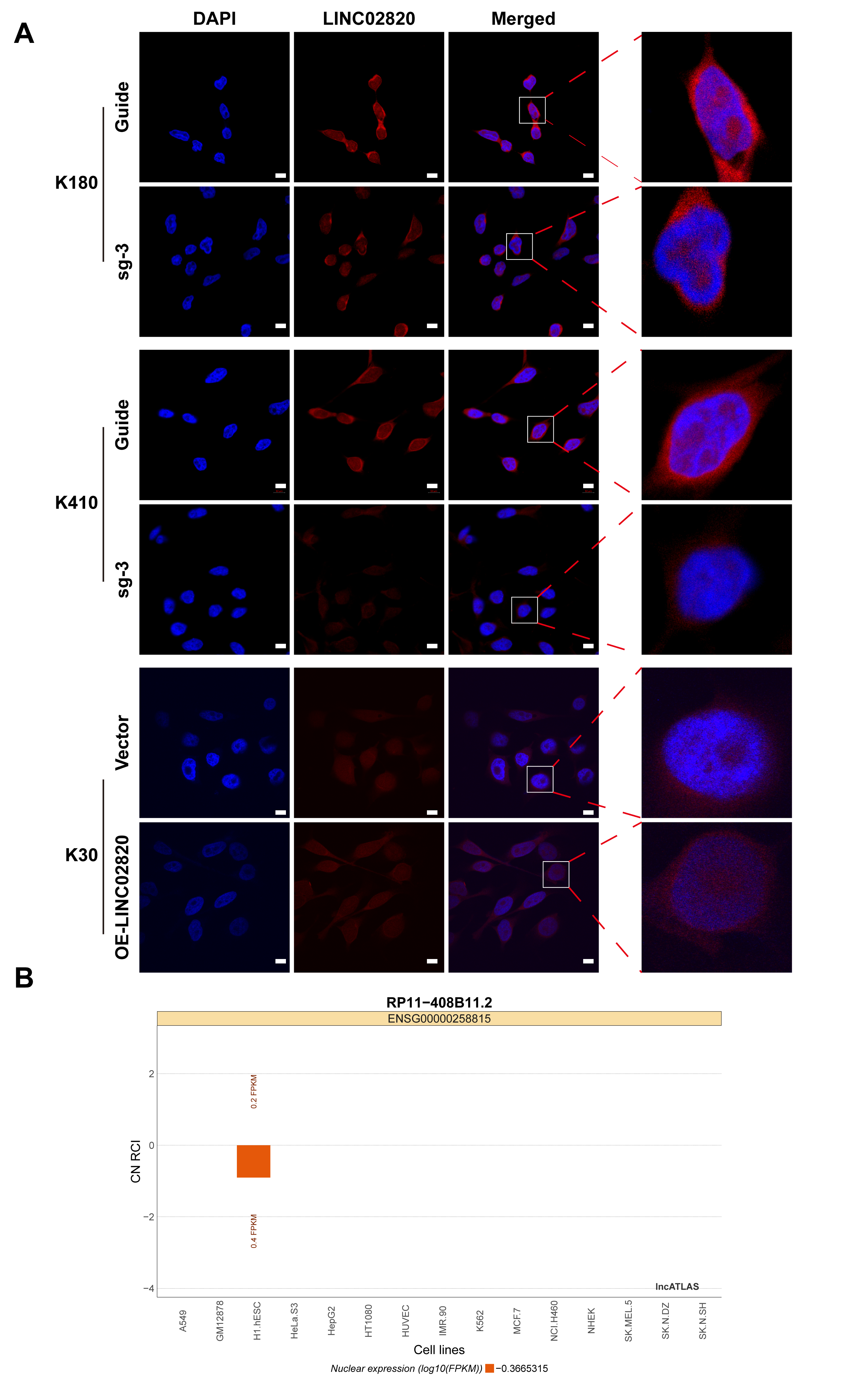

Supplement: Supplementary file 11 — supplementary figure-4 [file 41417_2022_554_MOESM11_ESM.tif]

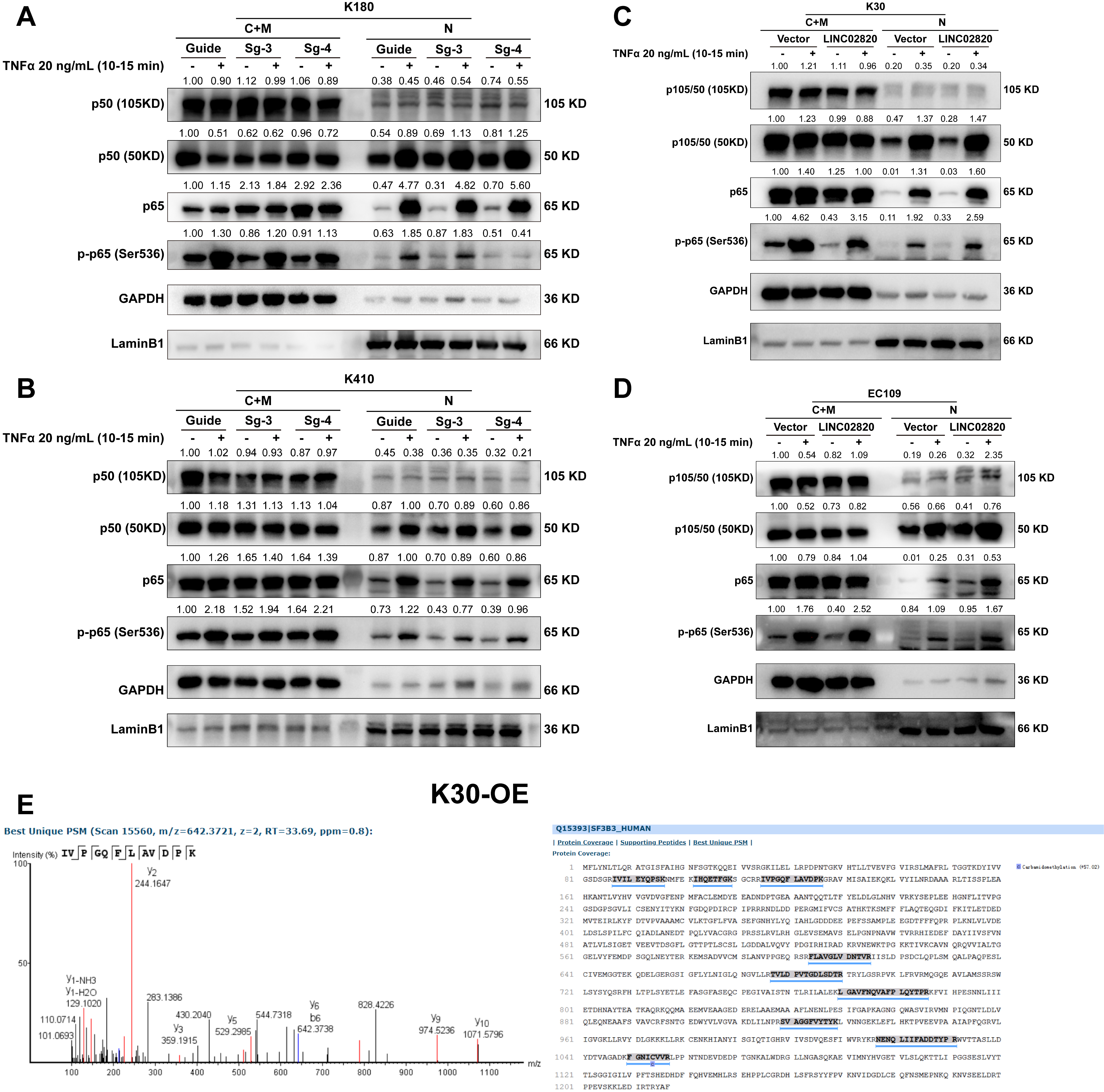

Supplement: Supplementary file 12 — supplementary figure-5 [file 41417_2022_554_MOESM12_ESM.tif]
