# Supplementary material for: Novel long noncoding RNA LINC02820 augments TNF signaling pathway to remodel cytoskeleton and potentiate metastasis in esophageal squamous cell carcinoma
Source: Cancer Gene Ther. 2022 Nov 10;30(2):375–87. doi: 10.1038/s41417-022-00554-2 (PMC9935391; doi:10.1038/s41417-022-00554-2)
Supplement: Supplementary file 13 — Supplementary Figures and Table Legends [file 41417_2022_554_MOESM13_ESM.docx]

**Supplementary Figure Legends**

**Supplementary Figure 1. LINC02820 Doesn’t Affect Proliferation but Promotes Metastasis in ESCC.**

(**A**) The copy number of LINC02820 per cell. (**B, C, D**) The statistical analysis of K180, K410, K30, and EC109 cells by colony formation experiment (“ns” means

no significant difference). (**E**) Analysis of the migration and invasion ability of K410 cells transfected with SiLINC02820 or siNC (scale bar, 100 μm). (**F**) Analysis of the migration and invasion ability of EC109 cells transfected with OE- LINC02820 or Vector (scale bar, 100 μm). (**G**) The migration and invasion ability of K410 cells transfected with LINC02820 suppression plasmid (sg-3, sg-4) or control plasmid (Guide) (scale bar, 100 μm).

**Supplementary Figure 2. Wound Healing Assay Showing that Inhibition or Over-expression of LINC02820 Affects ESCC Metastasis in Vitro.**

(**A, B, C**) Wound healing assay of K180 and K410 cells transfected with SiLINC02820 or siNC. (**D, E, F**) Wound healing assay of K30 and EC109 cells transfected with OE- LINC02820 or Vector. (**G, H**) Wound healing assay of K180 and K410 cells transfected with LINC02820 suppression plasmid (sg-3, sg-4) or control plasmid (Guide). *p < 0.05, **p < 0.01, ***p < 0.001.

**Supplementary Figure 3. LINC02820 Influence the Metastasis of ESCC Without Relying on EMT.**

(**A**)The E-cadherin and Vimentin when LINC02820 are inhibited or increased by immunofluorescence assay (scale bar, 50 μm).

**Supplementary Figure 4.** **LINC02820** **is mainly located in the Nucleus.**

(**A**) RNA-FISH was performed to determine LINC02820 in K180 and K410 cells transfected with LINC02820 suppression plasmid (sg-3) or control plasmid, and K30 cells transfected with OE- LINC02820 or Vector (scale bar, 10μm). (**B**) The lncATLAS (<https://lncatlas.crg.eu/>) predicts that the LINC02820 is located in the nucleus.

**Supplementary Figure 5.** **LINC02820 may Participate in TNF/NF-κB Signaling pathway.**

(**A**, **B, C, D**) Nuclear and cytoplasmic protein fractions were isolated from ESCC cell lines under TNFα (20 ng/mL). (**E**) Analysis of SF3B3 in K30 cells transfected with OE-LINC02820 by liquid chromatography-tandem mass spectrometry (LC-MS/MS).

**Supplementary Table Legends**

**Supplementary Table 1.** The Information of LINC02820 in Different Databases

**Supplementary Table 2.** The Data of Clinicopathological Parameters in 86 ESCC patients

**Supplementary Table 3.** The Information of ESCC Cell Lines

**Supplementary table 4.** The primers for RT-PCR

**Supplementary table 5.** The sequence of making Smart silencer RNA and sgRNA plasmids

**Supplementary Table 6:** The 27 differentially expressed lncRNAs in the three paired ESCC tissues and normal tissues

**Supplementary Table 7.** The pan-cancer analysis of LINC02820
